# When Suspicious Thyroid Cytology Isn’t Thyroid Cancer: Multinodular Goiter With Granulomatous Lymphadenopathy

**DOI:** 10.7759/cureus.114242

**Published:** 2026-08-09

**Authors:** Sharadha Sreenivasa, Jorge Lima Gallardo, Cheryl Gonzalez-Figueroa, Rachelle L Jean-Marie, Joseph S De Gaetano

**Affiliations:** 1 Family Medicine, Nova Southeastern University Dr. Kiran C. Patel College of Osteopathic Medicine, Fort Lauderdale, USA; 2 Family Medicine, Broward Health Medical Center, Fort Lauderdale, USA

**Keywords:** cervical lymphadenopathy, follicular neoplasm, granulomatous disease, non-necrotizing granulomatous inflammation, sarcoid-like reaction, sarcoidosis, thyroid malignancy mimic, thyroid nodule

## Abstract

Suspicious thyroid lesions are often evaluated using fine-needle aspiration (FNA) with the Bethesda System for Reporting Thyroid Cytopathology (TBSRCT) to estimate malignancy risk and guide management. Indeterminate findings create a diagnostic gray zone where neither molecular testing nor imaging alone can provide certainty. We report the case of a 57-year-old female with a progressively enlarging right-sided thyroid nodule, classified as Bethesda IV on FNA cytology, with a positive neuroblastoma rat sarcoma viral oncogene homolog (NRAS) mutation on ThyroSeq v3, suggestive of high-risk malignancy. Preoperative imaging also revealed suspicious cervical lymphadenopathy concerning for metastatic disease.

Given these findings, the patient underwent a total thyroidectomy. Final pathology demonstrated benign nodular hyperplasia with no evidence of carcinoma, revealing a false-positive molecular result. Notably, excised cervical lymph nodes revealed non-necrotizing granulomas, raising suspicion for sarcoidosis or other granulomatous diseases. Infectious and malignant etiologies were excluded. Subsequent evaluation with chest computed tomography (CT) confirmed pulmonary sarcoidosis, despite a normal serum angiotensin-converting enzyme (ACE) level.

This case highlights the diagnostic challenges of evaluating indeterminate thyroid nodules where radiographic, cytologic, and molecular findings may obscure the distinction between granulomatous inflammation and malignancy. This patient's presentation illustrates the importance of maintaining a broad differential diagnosis and using a multimodal approach to optimize clinical management and navigate diagnostic uncertainty.

## Introduction

Thyroid nodules are a common clinical finding, particularly among women, with a malignancy rate of approximately 7%-15% [1]. Evaluation of suspicious thyroid nodules is crucial to rule out thyroid cancer. However, identifying nodules that harbor malignancy remains a challenge, especially when standardized tests yield diagnostic gray zones. Fine-needle aspiration (FNA) and the Bethesda System for Reporting Thyroid Cytopathology (TBSRCT) are gold standards for determining and categorizing cancer risk and guiding management [1]. However, when a thyroid biopsy is categorized as Bethesda Category IV, an indeterminate finding that is suspicious for follicular neoplasm, cytology alone is not sufficient. Molecular testing like ThyroSeq v3 is integrated for further risk stratification, in the context of rat sarcoma (RAS) mutations, yielding a variable range of malignancy risk, anywhere from 40% to 80% [2]. Thus, high variability in disease classification and diagnostic gray zones necessitate a broader differential diagnosis that should include inflammatory pathologies that act as rare benign mimics of cancer [3].

Among the unexpected cancer mimics is granulomatous disease, characterized by chronic inflammatory reactions leading to formulation of granulomas. Granulomatous involvement of the thyroid is relatively uncommon but may manifest as nodules or gland enlargement. Granulomatous diseases such as sarcoidosis commonly affect the pulmonary system, forming non-necrotizing, non-caseating granulomas. Though it is a multisystem disease, thyroid involvement is rare, with a prevalence of 1%-4.5% among patients in autopsy studies, presenting as solitary nodules or diffuse goiter [3]. These granulomatous lesions can mirror thyroid malignancy on ultrasonography and molecular profiles. Thus, the underlying inflammatory process is often masked, limiting preoperative detection and often requiring identification from surgical pathology or autopsy [3]. This diagnostic ambiguity may lead to unnecessary surgical intervention in otherwise benign conditions. The high effectiveness of corticosteroids, immunosuppressants, and biologic agents in reducing the size of sarcoid masses further emphasizes the need for improved diagnostic tools to avoid surgical intervention [3].

We report a case of suspicious thyroid nodules with Bethesda IV cytology and positive molecular testing that ultimately showed benign thyroid pathology with an incidental finding of non-necrotizing granulomatous lymphadenitis upon surgical resection. This case highlights the challenges of evaluating highly suspicious thyroid nodules, the limitations of molecular testing, and the importance of considering granulomatous diseases, including sarcoidosis, as potential inflammatory mimics of malignancy.

## Case presentation

A 57-year-old female presented for evaluation of progressively enlarging right-sided thyroid nodules. Past medical history is significant for hypertension, protein C deficiency, prediabetes, hyperlipidemia, and deep vein thrombosis requiring inferior vena cava (IVC) filter placement in 2009, with ongoing rivaroxaban therapy. Her surgical history included a left thyroid lobectomy in 1997 for a benign left thyroid cyst, elective hysterectomy in 2010, and gastric lap-band placement in 2014.

Family history was significant for thromboembolic disease, including a daughter who passed away from a saddle pulmonary embolism and a son with recurrent deep vein thrombosis on anticoagulation. Multiple first-degree relatives have type 2 diabetes mellitus and hypertension; however, she denied a family history of thyroid malignancy or prior radiation exposure. Social history was negative for tobacco or illicit drug use, and the patient reported occasional social alcohol consumption. 

Diagnostic course

**Figure 1:**
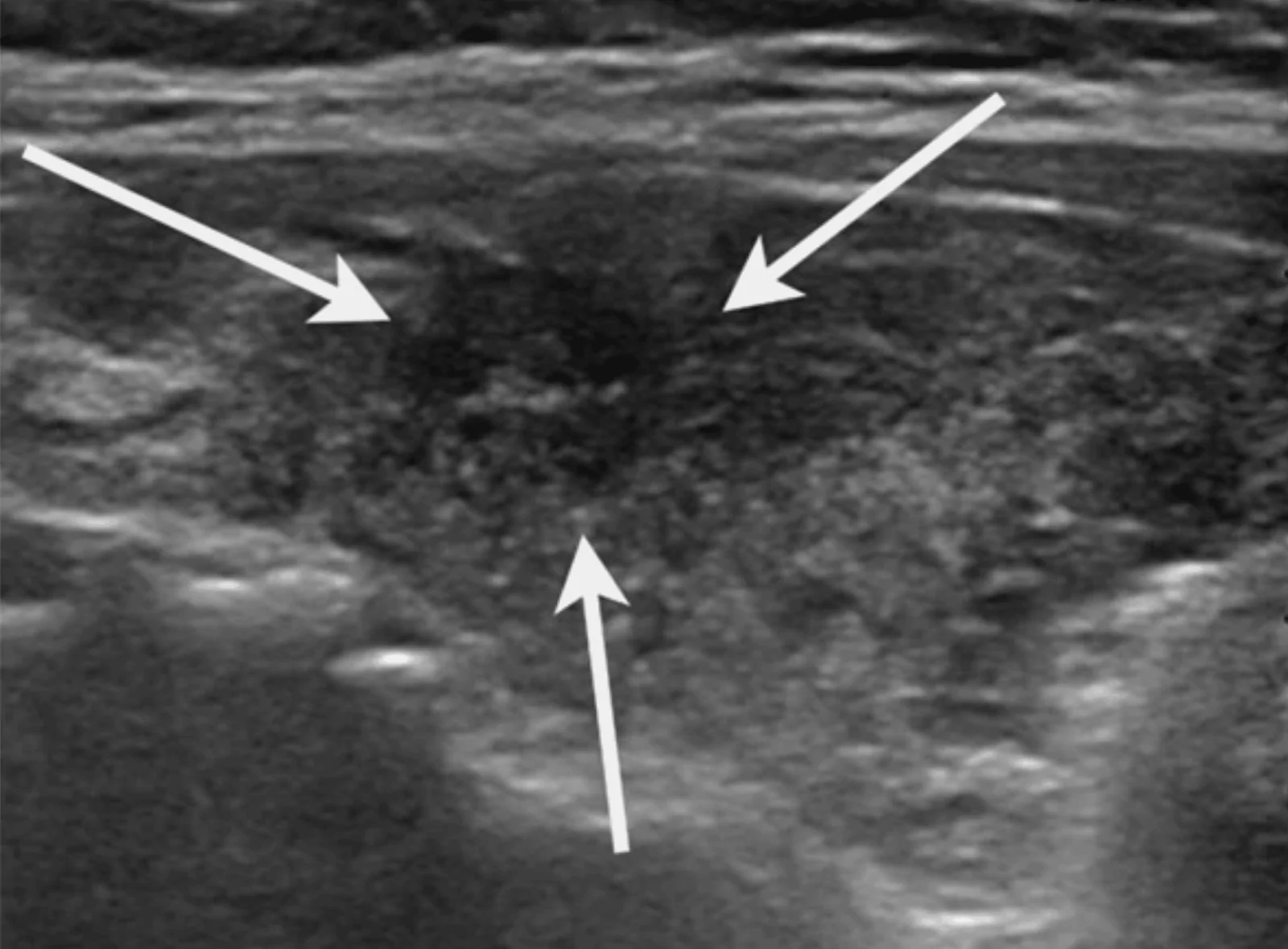
Heterogeneous thyroid with hypoechoic nodule. Longitudinal ultrasound image showing an irregular, hypoechoic nodule (white arrows) within a heterogeneous background of the thyroid lobe. Credit: Adapted from Park et al. [4], BMC Cancer. Used under the Creative Commons Attribution (CC BY) 2.0 International License, with cropping.

The patient underwent serial thyroid ultrasounds from 2019 to 2025, which demonstrated a progressively enlarging, heterogeneous right thyroid lobe (5.3-5.8 cm) with multiple solid and complex nodules, resembling the sonographic appearance depicted in Figure 1. There were no microcalcifications, suspicious vascular features, or pathologic lymphadenopathy identified. In 2020, FNA of the right-sided nodules revealed benign cytology (Bethesda II) findings. At that time, endocrinology recommended clinical monitoring rather than continued serial imaging, unless the patient developed compressive symptoms such as dysphagia, cough, and shortness of breath. The patient was also evaluated by a rheumatologist in 2022 following the finding of a positive antinuclear antibody (ANA) at a titer of 1:40. However, there was no serologic or clinical evidence of antiphospholipid syndrome or systemic autoimmune disease. The patient remained clinically asymptomatic and denied cough, shortness of breath, blurry vision, or skin lesions. 

Cytology and Molecular Testing

A 2024 thyroid ultrasound demonstrated interval growth of a 2.9 cm complex nodule in the right mid-lobe. A repeat FNA performed in 2025 showed findings of Bethesda Category IV cytology, suspicious for follicular neoplasm. Subsequent ThyroSeq v3 molecular testing was positive for a neuroblastoma rat sarcoma viral oncogene homolog (NRAS) mutation with 40% allele frequency, along with positive copy number and gene expression alterations, demonstrating an estimated malignancy risk of 70%. These findings were associated with follicular patterned carcinoma or non-invasive follicular thyroid neoplasm with papillary-like nuclear features (NIFTP). The remaining nodules were benign clonal follicular adenomas. Based on the high molecular risk, complete thyroidectomy was recommended.

Preoperative Lymph Node Evaluation

In 2025, preoperative flexible laryngoscopy revealed normal vocal cord mobility and no abnormalities. A repeat thyroid and neck ultrasound was performed during this surgical consultation, which identified prominent cervical lymph nodes in the right level IV and bilateral level VI compartments, measuring up to 1.8 cm, demonstrating increased hilar vascularity. Additional abnormal lymph nodes were also identified adjacent to the thyroid and within the bilateral lateral neck compartments. To rule out malignancy, subsequent ultrasound-guided FNA of the right cervical lymph node was performed. Cytology was negative for ancillary stains acid-fast bacilli (AFB) and Grocott methenamine silver (GMS) and cytokeratin AE1/AE3 staining, arguing against infectious and epithelial malignancy etiologies. 

Preoperative thyroid mapping was performed, and a chest radiograph was not obtained due to a low clinical suspicion for sarcoidosis. The complete blood count and comprehensive metabolic panel were within normal limits, including a serum calcium level of 9.9 mg/dL. Thyroid-stimulating hormone level was also within the normal range at 2.33 mlU/L.

Surgical Management and Pathology

The patient underwent a total thyroidectomy in October 2025. Final surgical pathology revealed benign nodular hyperplasia of the right thyroid lobe, with no evidence of carcinoma, demonstrating a false-positive molecular result. However, evaluation of the excised cervical lymph nodes revealed multiple well-formed, non-necrotizing granulomas. AFB and GMS stains remained negative. In the absence of infection or malignancy, these incidental findings raised strong suspicion for non-necrotizing granulomatous disease such as sarcoidosis manifesting as granulomatous lymphadenitis.

Postoperative Course

The patient was started on levothyroxine 100 mcg daily and reported improving fatigue with intermittent mild hoarseness. The patient denied heat or cold intolerance, mood changes, palpitations, or compressive symptoms. The surgical incision healed without complications and the patient remained clinically stable. Given the incidental finding of non-necrotizing granulomas, the patient was referred to pulmonology and rheumatology to evaluate for systemic sarcoidosis or other granulomatous disease. Rheumatology recommended follow-up on an as-needed basis contingent upon the development of new symptoms. 

**Figure 2:**
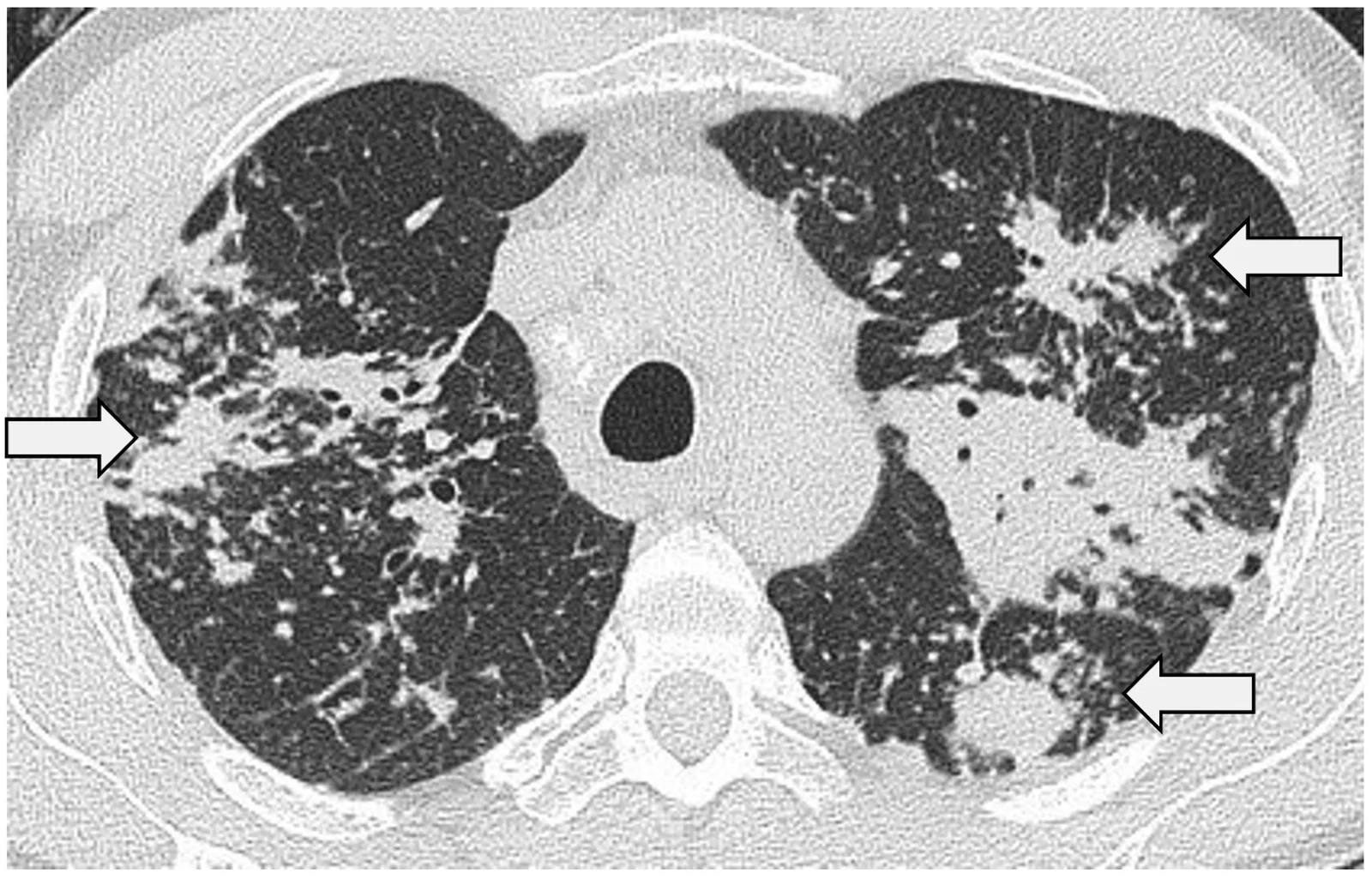
Radiographic findings of pulmonary sarcoidosis. Chest CT demonstrating bilateral upper lobe pulmonary nodules with surrounding clusters of micronodules (white arrows), findings consistent with sarcoidosis. Credit: Image adapted from Bailey et al. [5], J Clin Med. Used under the CC BY 4.0. CT, computed tomography

In 2026, pulmonology evaluation and subsequent diagnostic workup included a non-contrast computed tomography (CT) of the chest, which showed mediastinal and bilateral hilar lymphadenopathy, including the subcarinal and paratracheal lymph nodes measuring 1.1-1.3 cm. The scan also showed multiple clusters of 2-4 mm perilymphatic solid nodules predominantly involving the upper lobes, along with a few scattered bilateral ground-glass nodules. These findings were consistent with Stage II pulmonary sarcoidosis. There was no evidence of axillary lymphadenopathy, emphysema, bronchiectasis, or chronic interstitial lung disease. Overall, these findings were similar to those demonstrated on the CT chest shown in Figure 2. 

Laboratory evaluation showed a normal serum angiotensin-converting enzyme (ACE) level of 31 U/mL. Pulmonary function testing demonstrated mild small airways disease without evidence of restrictive lung disease or impaired gas exchange. The patient is awaiting outpatient follow-up for management of pulmonary sarcoidosis.

## Discussion

Granulomatous inflammatory diseases represent a broad group of conditions characterized by chronic immune activation leading to the formation of granulomas. The spectrum of granulomatous disease is wide and includes infectious etiologies such as mycobacterial or fungal infections, sarcoid-like reactions to malignancy, neoplastic processes, foreign body reactions, and systemic diseases [6]. Among these, non-necrotizing granulomas are most associated with sarcoidosis [7]. Systemic sarcoidosis most commonly presents with pulmonary involvement in 90% of patients, with cough, wheezing, and shortness of breath, while cutaneous and ocular manifestations are seen in 20%-30% of cases, with skin lesions and visual disturbances [8]. Thyroid sarcoidosis has been reported in as few as 1%-4% of patients and most commonly presents as an asymptomatic goiter, although compressive symptoms such as dyspnea or dysphagia can occur, along with possible thyroid dysfunction or cervical lymphadenopathy [3]. In patients with thyroid nodules, the presence of enlarged cervical lymph nodes, particularly in the lateral neck compartments, raises further concern for metastatic spread from primary thyroid cancer [9]. As in our case, multinodular goiters can produce indeterminate cytology, creating a significant diagnostic challenge. 

Sarcoid-like reactions are also associated with malignancy, where noncaseating granulomas develop in tumor-draining lymph nodes, without evidence of systemic sarcoidosis [10]. Although sarcoid-like reactions have been reported with various malignancies, very few cases of sarcoid reactions have been associated with thyroid cancer, particularly metastatic papillary thyroid carcinoma [11]. Thus, the distinction between granulomatous diseases and malignancy is challenging as conditions overlap or have variant manifestations. Therefore, the findings of granulomatous lymphadenopathy should warrant a comprehensive evaluation.

**Figure 3:**
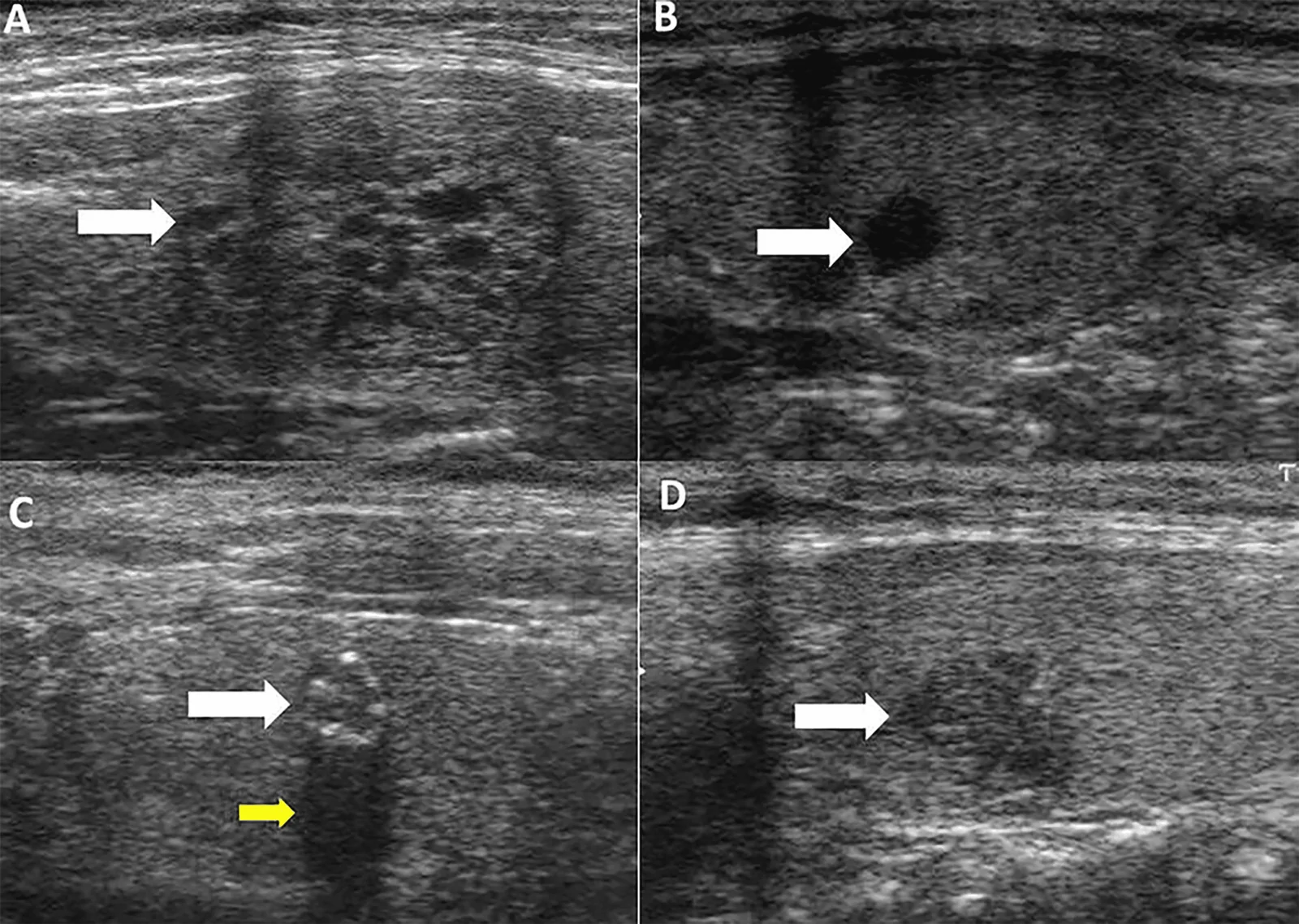
Sonographic features of benign and suspicious thyroid nodules. (A-B) These images depict likely benign nodules, including a well-defined isoechoic nodule (white arrow, A) and a well-defined anechoic cystic nodule (white arrow, B). (C-D) These images depict nodules with suspicious features, including a well-defined hypoechoic solid nodule with calcifications (white arrow, C) and posterior acoustic shadowing (yellow arrow, C), as well as an irregular hypoechoic solid nodule (white arrow, D). Credit: Adapted from Sultan [14], Cureus. Used under CC BY 4.0.

**Figure 4:**
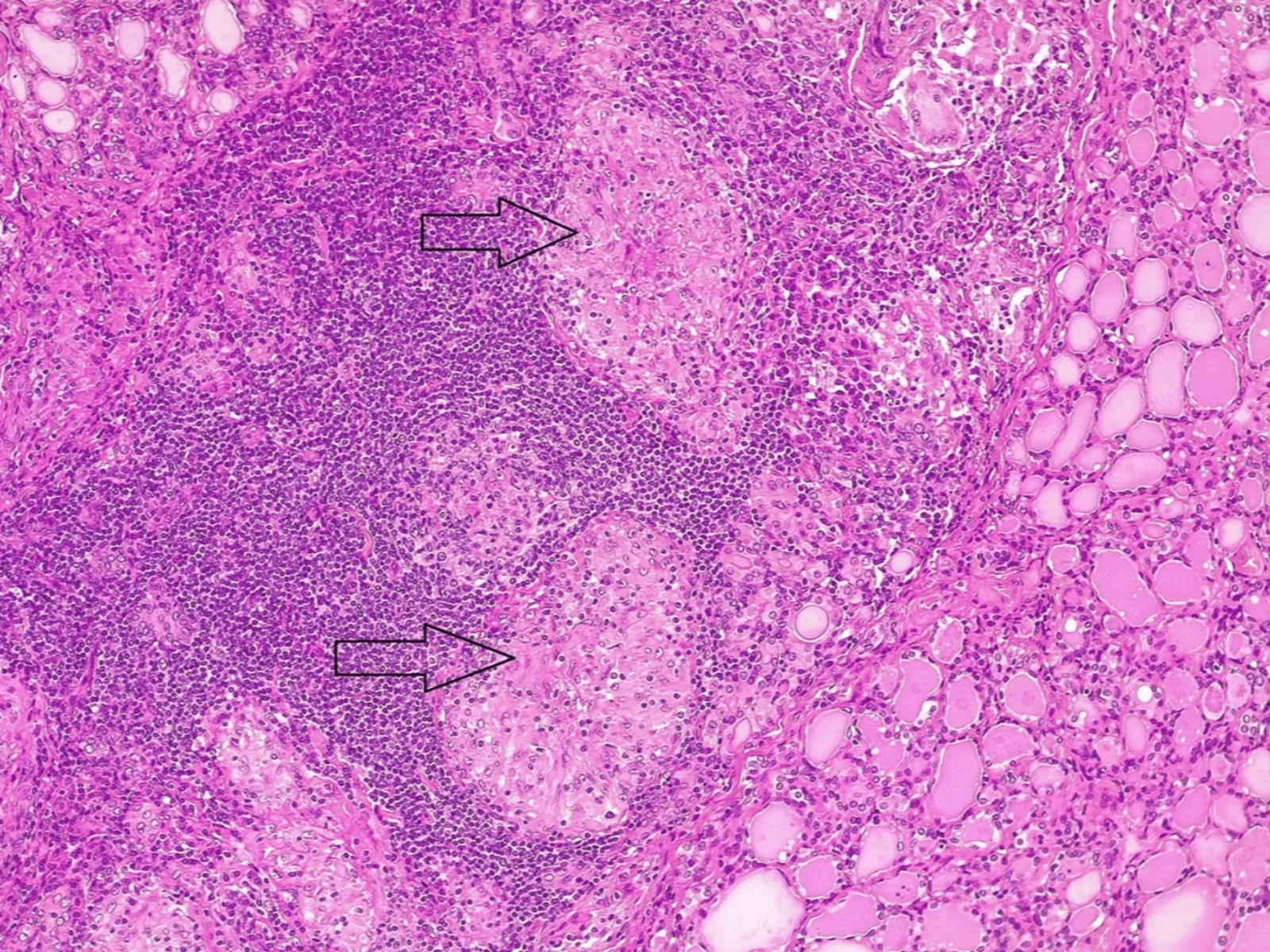
Non-necrotizing granulomas of the thyroid. Thyroid histopathology demonstrating multiple non-necrotizing granulomas (black arrows), a characteristic histologic finding in sarcoidosis. Credit: Adapted from Salih et al. [15], Acta-Oto Laryngol Case Rep. Used under CC BY 4.0, with cropping.

Imaging and molecular testing can help improve estimation of malignancy risk but often do not provide diagnostic certainty. Molecular classifiers, such as ThyroSeq v3, are useful for risk stratification of indeterminate cytology. However, the probability of malignancy among ThyroSeq v3-positive cases varies widely, ranging from 30% to 99%, in atypical cytologic categories [2]. In our case, a ThyroSeq v3 result estimated a 70% risk of malignancy, but final pathology revealed a different diagnostic conclusion. Diagnostic ambiguity also persists with imaging modalities, as both sarcoidosis and sarcoid-like reactions have been shown to have hypermetabolic activity or enlarged lymph nodes on ultrasound, CT, and positron emission tomography (PET) scans, closely mimicking metastatic thyroid cancer [12,13]. Representative ultrasound images of thyroid nodules are shown in Figures 3A-3D, demonstrating a spectrum of thyroid nodule characteristics, ranging from well-defined benign patterns to suspicious features associated with increased risk of malignancy. In these cases, definitive diagnoses are often only reached after tissue biopsy, with histopathology demonstrating noncaseating granulomas, as shown in Figure 4. It is important to highlight the history of granulomatous diseases mimicking thyroid carcinoma, particularly with cervical lymphadenopathy. Thus, granulomatous diseases including sarcoidosis cannot be excluded. 

Additionally, cases of coexisting sarcoidosis and thyroid carcinoma have been reported, adding another layer of complexity [16]. In such scenarios, identifying systemic findings, including pulmonary involvement or elevated serum markers such as ACE levels, ANA, serum calcium, and 25-hydroxy-vitamin D3, may aid in diagnosis [17]. Serum ACE levels may exceed twice the upper limit of normal in systemic sarcoidosis, offering a diagnostic specificity as high as 93% [18]. However, these adjunctive findings are only present in 30%-80% of patients with sarcoidosis, and no specific biomarker has been identified to date that reliably confirms the diagnosis [17,18]. Given our patient's normal serum ACE level, it further highlights the limitation of ACE as a diagnostic adjunct, as normal levels do not exclude the presence of sarcoidosis, further complicating diagnostic accuracy. Similarly, studies using endobronchial ultrasound-guided biopsy to evaluate granulomatous lymphadenitis reveal that not all cases ultimately represent sarcoidosis [19]. Therefore, histopathologic evaluation following a surgical resection is often the definitive method. For distinguishing these entities, identification of capsular or vascular invasion is required to distinguish benign follicular adenoma and follicular carcinoma [20]. In our patient, despite high-risk molecular findings, final pathology revealed benign disease. Therefore, it raises the question of how to integrate and expand clinical investigations to increase scope and potential for rarer differential conditions. 

Approaches to managing these conditions differ considerably. Metastatic thyroid cancer may require oncologic evaluation, hormone suppression therapy, radioactive iodine therapy, or surgical intervention [21]. In contrast, benign granulomatous diseases such as sarcoidosis are often managed conservatively with corticosteroids or observation depending on the extent of systemic involvement [22], while sarcoid-like reactions are generally managed in the context of the underlying malignancy. Thus, distinguishing between metastatic and granulomatous disease is crucial for proper treatment. 

Key distinguishing features between thyroid malignancy and granulomatous disease are summarized in Table 1, emphasizing the importance of integrating clinical presentation, imaging, cytologic findings, and histopathologic evaluation when approaching similar complex cases.

**Table 1:** Diagnostic features distinguishing thyroid malignancy from granulomatous disease in the evaluation of suspicious thyroid nodules. Bethesda, Bethesda System for Reporting Thyroid Cytopathology; US, ultrasonography; CT, computed tomography; PET, positron emission tomography

Features	Suggestive of Thyroid Malignancy	Suggestive of Granulomatous Disease (Sarcoidosis)
Clinical Presentation	Thyroid nodule with or without compressive symptoms. Cervical lymph nodes, especially in lateral neck compartments, may indicate metastatic spread from thyroid carcinoma [9].	Often asymptomatic but may present with goiter, compressive symptoms, thyroid dysfunction, or cervical lymphadenopathy [3]. Pulmonary, cutaneous, and ocular involvement suggest systemic sarcoidosis [8].
Imaging	Suspicious US features include hypoechoic nodules, irregular margins, microcalcifications, and possible extrathyroidal extension [14].	Thyroid involvement is uncommon, and US, CT, PET imaging may demonstrate enlarged or hypermetabolic nodules [12,13]. Bilateral hilar lymphadenopathy on chest imaging is a characteristic finding [5].
Fine-Needle Aspiration	Bethesda V (suspicious for malignancy) or Bethesda VI (malignant) strongly suggest thyroid carcinoma [1]. Bethesda III/IV cytology is indeterminate and often requires molecular testing for risk stratification [1,2].	May show atypical or indeterminate Bethesda III/ IV cytology due to inflammatory changes, requiring histopathologic evaluation and exclusion of other etiologies [1,3].
Pathology	Follicular thyroid carcinoma demonstrates capsular or vascular invasion [20].	Multiple non-necrotizing granulomas are characteristic findings [15].
Management	Management is guided by pathology, staging, and risk stratification, possibly requiring thyroidectomy, radioactive iodine therapy, thyroid hormone suppression, and oncologic follow-up [21].	Management is often conservative or involves corticosteroids, immunosuppressants, or biologics, depending on the extent of systemic involvement [3,22].

## Conclusions

Overall, this case highlights the diagnostic challenges clinicians face when granulomatous disease presents with clinical features resembling malignancy. When clinical, radiographic, and cytologic findings are unclear, recognizing uncommon causes can be valuable in guiding management and refining complex diagnoses. Future research should focus on improving diagnostic accuracy through methods such as refining molecular testing or identifying features that better distinguish malignancy from inflammatory mimics. These efforts may provide a broader tool set for clinicians to craft wider differentials for such cases. Thus, a multidisciplinary and flexible approach is essential when evaluating patients with concurrent thyroid nodules and granulomatous lymphadenopathy.
